# High-resolution matrix-assisted laser desorption ionization–imaging mass spectrometry of lipids in rodent optic nerve tissue

**Published:** 2013-03-19

**Authors:** David M. G. Anderson, Daniel Mills, Jeffrey Spraggins, Wendi S. Lambert, David J. Calkins, Kevin L. Schey

**Affiliations:** 1 Department of Biochemistry, Vanderbilt University School of Medicine, Nashville, TN; 2 Mass Spectrometry Research Center, Vanderbilt University School of Medicine, Nashville, TN; 3 Department of Ophthalmology and Visual Sciences, Vanderbilt University School of Medicine, Nashville, TN

## Abstract

**Purpose:**

To develop a method for generating high spatial resolution (10 µm) matrix-assisted laser desorption ionization (MALDI) images of lipids in rodent optic nerve tissue.

**Methods:**

Ice-embedded optic nerve tissue from rats and mice were cryosectioned across the coronal and sagittal axes of the nerve fiber. Sections were thaw mounted on gold-coated MALDI plates and were washed with ammonium acetate to remove biologic salts before being coated in 2,5-dihydroxybenzoic acid by sublimation. MALDI images were generated in positive and negative ion modes at 10 µm spatial resolution. Lipid identification was performed with a high mass resolution Fourier transform ion cyclotron resonance mass spectrometer.

**Results:**

Several lipid species were observed with high signal intensity in MALDI images of optic nerve tissue. Several lipids were localized to specific structures including in the meninges surrounding the optic nerve and in the central neuronal tissue. Specifically, phosphatidylcholine species were observed throughout the nerve tissue in positive ion mode while sulfatide species were observed in high abundance in the meninges surrounding the optic nerve in negative ion mode. Accurate mass measurements and fragmentation using sustained off-resonance irradiation with a high mass resolution Fourier transform ion cyclotron resonance mass spectrometer instrument allowed for identification of lipid species present in the small structure of the optic nerve directly from tissue sections.

**Conclusions:**

An optimized sample preparation method provides excellent sensitivity for lipid species present within optic nerve tissue. This allowed the laser spot size and fluence to be reduced to obtain a high spatial resolution of 10 µm. This new imaging modality can now be applied to determine spatial and molecular changes in optic nerve tissue with disease.

## Introduction

The optic nerve is the conduit for transmitting visual signals along retinal ganglion cell axons to multiple sub-cortical nuclei involved in various vision-related tasks [1]. These axons are unmyelinated in the retina and remain so in exiting the retina through the optic disc until passing through the optic nerve head [2]. Similar to other nerves within the central nervous system, the optic nerve has associated meninges, including the arachnoidal and pial membranes and the dura, which blends into the sclera. Beyond the nerve head, the fibers become myelinated by oligodendrocytes [3] similar to the white matter in brain and spinal cord tissue [4]. Lipids are a major component of myelin and meninges and support the functional activity of neuronal tissue; therefore, the lipid composition and spatial distribution of lipids in tissues are key to normal nerve function [5]. Optic nerve lipid composition studies indicate that major lipid components include neutral lipid species such as cholesterol, glycosphingolipids such as cerebrosides, and several glycerophospholipids [5,6]. Changes in optic nerve structure and function are key contributors to glaucoma, the most common optic neuropathy, and include early insult to ganglion cell axons [7]. Many pathogenic features of glaucoma involve spatially specific changes in nerve function and morphology [8,9]. Thus, understanding the spatial pattern of molecular changes is essential for probing molecular mechanisms of disease and identifying new therapeutic targets.

Matrix-assisted laser desorption ionization (MALDI) imaging mass spectrometry (IMS) is a novel imaging modality capable of providing molecular ion maps across tissue surfaces including maps of lipids, proteins, and metabolites [10-12]. In a MALDI-IMS experiment, information about molecular distribution and relative intensity is acquired. In recent years, several papers on imaging mass spectrometry of ocular tissue, including lens, lens capsule, lens lipids, flat mounted retina, and retinal cross sections, have been published [13-19]. Since MALDI-IMS was developed in 1997 by the Caprioli group [20], the technology has progressed rapidly to allow for greater sensitivity and improved spatial resolution. The spatial resolution of MALDI-IMS is currently limited by several factors: laser spot size, sensitivity (which is affected by area irradiated and laser power [21]), matrix coverage, and analyte delocalization on the sample surface.

Several recent publications have demonstrated high spatial resolution mass spectrometry imaging of fatty acids, lipids, and proteins with varying sample preparation methods [19,22-25]. Matrix application by sublimation was first shown in 2007 [26] to provide even coverage of matrix over the sample surface without the need for solvents and to produce high-intensity signals for lipid analysis. Washing protocols for tissue sections are an important step in MALDI-IMS sample preparation to remove biologic salts and endogenous compounds within tissue sections; however, washing protocols are not always suitable for lipid analysis due to potential delocalization and removal of lipids. To improve sensitivity toward lipid analysis using MALDI-IMS by reducing the formation of sodium and potassium adducts, ammonium acetate has been shown to improve the mass spectra acquired and the MALDI images generated [27,28].The data presented here demonstrate the MALDI-IMS potential for optic nerve imaging, by imaging mouse and rat optic nerves at high spatial resolution (10 µm) to identify lipid spatial distributions and relative abundance in the tissue.

## Methods

This study was conducted in accordance with regulations set forth in the Association for Research in Vision and Ophthalmology Statement for the Use of Animals in Ophthalmic and Vision Research. Animal protocols were approved by the Institutional Animal Care and Use Committee of the Vanderbilt University Medical Center in accordance with guidelines published in the US Public Health Service Policy on Humane Care and Use of Laboratory Animals.

Whole eyes with the optic nerve attached were removed from Brown Norway rats and C57 mice following cervical dislocation and rapidly frozen in deionized water by placing the eye tissue into a cup fashioned from parafilm that was floated in a weighing boat on top of liquid nitrogen in a polystyrene container. Care was taken to orient the optic nerve so that it could be sectioned along the sagittal axis of the nerve. The ice-embedded sample was then mounted using Tissue-Tek OCT compound (Torrance, CA). Twelve-micron sections were cut with a Leica CM3050S cryostat (Wetzlar, Germany) and thaw mounted onto AB Sciex (Concord, Canada) gold-coated MALDI target plates. The sections were dehydrated in a vacuum desiccator overnight and then washed three times for 30 s each with 100 mM ammonium acetate (Sigma-Aldrich, St. Louis, MO) before being dried again for another 2 h. 2,5-Dihydroxybenzoic acid (DHB; Sigma-Aldrich) was deposited by sublimation onto the samples using a custom sublimation apparatus for 20 min at 54 mTorr pressure at 120 °C. The region containing the optic nerve was imaged in positive and negative ion modes for mouse tissue and positive ion mode for rat tissue using a MALDI-TOF (time-of-flight) mass spectrometer (UltrafleXtreme II, Bruker Daltonics, Billerica, MA) equipped with a Smartbeam II 1 kHz Nd:YAG frequency tripled to 355 nm wavelength. The laser was set to the minimum spot size with 8% laser power. External calibration was performed with a series of phosphorus clusters before imaging data were acquired [29], and internal calibration, based on masses identified with Fourier transform ion cyclotron resonance (FT-ICR), was performed after the data were acquired to ensure the best mass accuracy from the TOF data. Images were obtained with a spatial resolution of 10×10 µm with 100 laser shots per pixel. Images were generated using Bruker FlexImaging 2.1 software, and the data were normalized to the total ion current.

Lipid identification from the mouse optic nerve tissue was performed using information from two experiments: 1) accurate mass profile scans and 2) tandem mass spectrometry. Both experiments were conducted on tissue sections taken adjacent to those imaged. These data were collected using a MALDI FT-ICR mass spectrometer (9.4 Tesla Apex-Qe, Bruker Daltonics) equipped with an Apollo II dual ion source and the same 355 nm solid-state laser as described above. External calibration was done using a series of phosphorus clusters (29; P_n_, n=5–65) resulting in mass accuracies of better than 2 ppm for all analytes. Tandem mass spectrometry experiments were performed using a combination of continuous accumulation of selected ions (CASI) and sustained off-resonance irradiation collision-induced dissociation (SORI-CID) [30]. CASI (50 laser shots/fill, approximately 100 CASI fills) was used to increase the signal intensities of the mass selected precursor ions in a linear quadrupole (1.5 Da selection window) in the source region of the instrument before fragmentation in the ICR cell. Fragmentation was induced with SORI-CID (pulsed argon, frequency offset: 500 Hz, SORI time: 0.25 s) with a range of SORI power (0.45% to 0.75%) to maximize the number of fragment ions. To promote more structurally informative fragmentation data, 0.2 µl of lithium chloride (100 mM) was pipetted onto the DHB-coated tissue surface of one section for positive ion analysis [31,32], and 0.2 µl of aniline (65 mM) was pipetted onto the sublimed DHB for negative ion analysis. Spectral interpretation was accomplished using LIPID Metabolites And Pathways Strategy (LIPID MAPS) to match the accurate mass of the precursor ion and manually by interpreting the fragmentation patterns.

## Results

**Figure 1:**
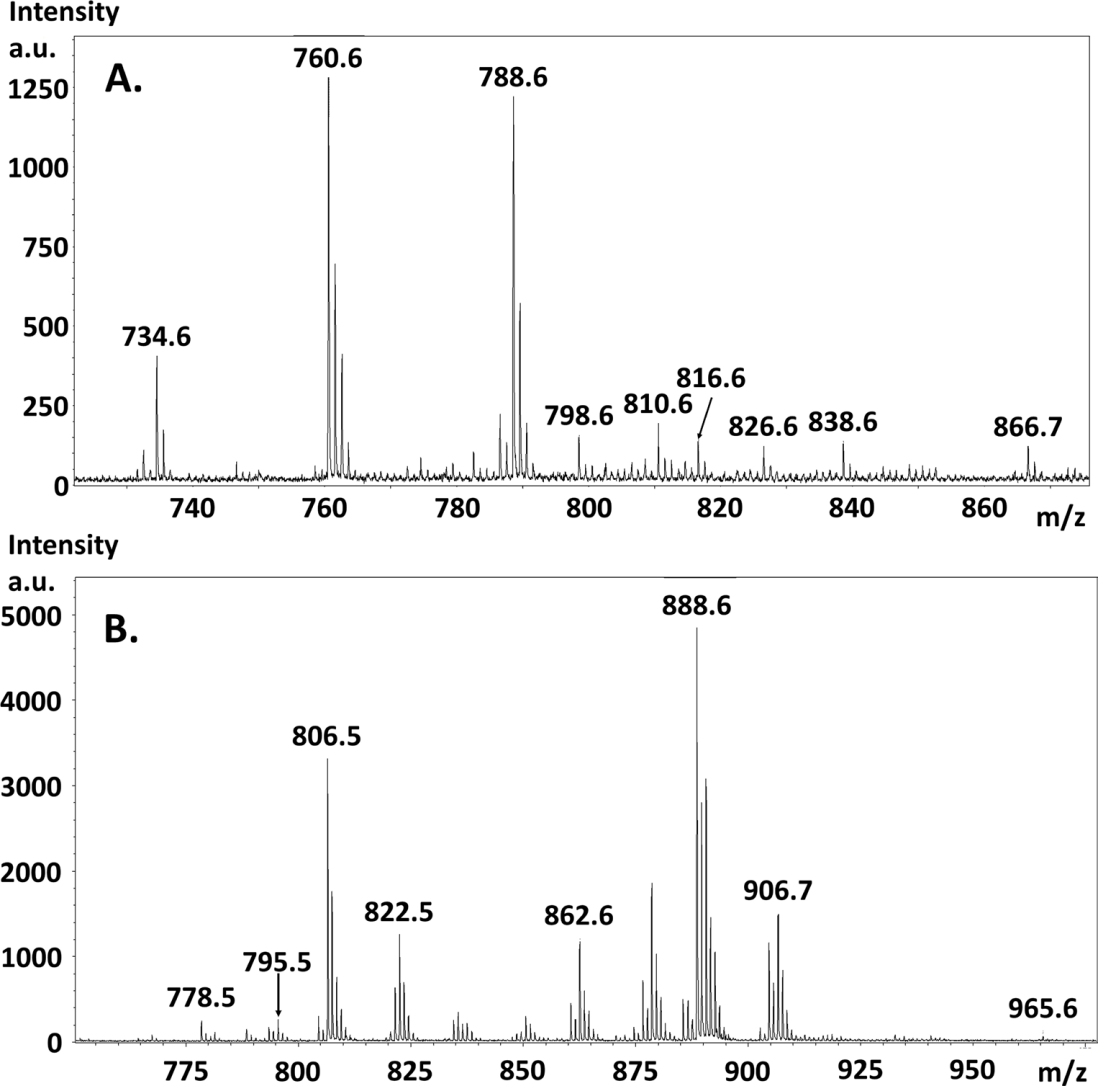
Matrix-assisted laser desorption ionization time-of-flight mass spectra from optic nerve tissue. **A**: Positive ion mode matrix-assisted laser desorption ionization–time-of-flight (MALDI-TOF) mass spectrum from mouse optic nerve tissue showing numerous peaks in the m/z 700–875 region. **B**: Negative ion mode MALDI-TOF mass spectrum from mouse optic nerve tissue showing numerous peaks in the m/z 760–940 region.

Intense signals were obtained from the meninges surrounding the optic nerve and from the internal nerve tissue using the sublimated DHB matrix. Figure 1A shows a positive ion MALDI mass spectrum from mouse optic nerve tissue with numerous peaks in the m/z 700–875 region. Figure 1B shows a negative ion MALDI mass spectrum from mouse optic nerve tissue with numerous peaks in the m/z 760–940 region. Table 1 lists the lipid signals assigned a structure based on accurate mass measurement and comparison to the LIPID MAPS database. Appendix 1 shows the spatial localization of these lipids within the optic nerve sections. Table 2 lists the lipid signals observed in negative ion mode assigned a structure based on accurate mass measurement and comparison to the LIPDS MAPS database. Appendix 2 shows the spatial localization of these lipids within the optic nerve sections.

**Table 1:** Optic nerve lipids observed in positive ion mode and structures assigned based on mass accuracy.

Observed m/z	Theoretical m/z	ppm error	Identification^a^	Molecular Formula
496.3407	496.3398	−1.89	LPC(16:0/0:0)	C_24_H_51_NO_7_P+
524.3708	524.3711	0.58	LPC(18:0/0:0)	C_26_H_55_NO_7_P+
732.5545	732.5538	−0.95	PC(32:1)	C_40_H_79_NO_8_P+
734.5690	734.5694	0.65	PC(32:0)	C_40_H_81_NO_8_P++
758.5691	758.5694	0.43	PC(34:2)	C_42_H_81_NO_8_P+
760.5850	760.5851	0.12	PC(34:1)	C_42_H_83_ NO_8_P+
782.5690	782.5694	0.57	PC(36:4)	C_44_H_81_NO_8_P+
786.6007	786.6007	−0.01	PC(36:2)	C_44_H_85_NO_8_P+
788.6161	788.6164	0.42	PC(36:1)	C_44_H_87_NO_8_P+
806.5709	806.5694	−1.85	PC(38:6)	C_46_H_81_NO_8_P+
808.5852	808.5851	−0.19	PC(38:5)	C_46_H_83_NO_8_P+
810.6009	810.6007	−0.15	PC(38:4)	C_46_H_85_NO_8_P+
813.6851	813.6844	−0.90	SM(d18:1/24:1)	C_47_H_94_N_2_O_6_P+
832.5863	832.5851	−1.48	PC(40:7)	C_48_H_83_NO_8_P+
834.6000	834.6007	0.83	PC(40:6)	C_48_H_85_NO_8_P+
842.6632	842.6633	0.16	PC(40:2)	C_48_H_93_NO_8_P+
844.6784	844.6790	0.69	PC(40:1)	C_48_H_95_NO_8_P+
853.7277	853.7280	0.29	TG(52:5)	C_55_H_97_O_6_+
855.7419	855.7436	1.99	TG(52:4)	C_55_H_99_O_6_+
870.6948	870.6946	−0.18	PC(42:2)	C_50_H_97_NO_8_P+
879.7439	879.7436	−0.30	TG(54:6)	C_57_H_99_O_6_+
881.7593	881.7593	0.01	TG(54:5)	C_57_H_101_O_6_+
892.6784	892.6790	0.66	PC(44:5)	C_52_H_95_NO_8_P+
907.7738	907.7749	1.23	TG(56:6)	C_59_H_103_O_6_+

^a^ Identifications made by mass accuracy alone

**Table 2:** Optic nerve lipids observed in negative ion mode and structures assigned based on mass accuracy.

Observed m/z	Theoretical m/z	ppm error	Identification^a^	Molecular Formula
778.5148	778.5145	−0.44	SulfoHex-Cer(d18:1/16:0)	C_40_H_76_NO_11_-
788.5449	788.5447	−0.27	PS(36:1)	C_42_H_79_NO_10_P-
806.5457	806.5458	0.02	SulfoHex-Cer(d18:1/18:0)	C_42_H_80_NO_11_-
810.5299	810.5291	−1.07	PS(38:4)	C_44_H_77_NO_10_P-
812.545	812.5447	−0.35	PS(38:3)	C_44_H_79_NO_10_P-
816.5745	816.576	1.86	PS(38:1)	C_44_H_83_NO_10_P-
834.5292	834.5291	−0.22	PS(40:6)	C_46_H_77_NO_10_P-
834.5773	834.5771	−0.24	SulfoHex-Cer(d18:1/20:0)	C_44_H_84_NO_11_-
838.5613	838.5604	−1.1	PS(40:4)	C_46_H_81_NO_10_P-
857.5187	857.5186	−0.2	PI(36:4)	C_45_H_78_O_13_P-
862.6081	862.6084	0.25	SulfoHex-Cer(d18:1/22:0)	C_46_H_88_NO_11_-
878.6028	878.6033	0.51	SulfoHex- Cer(d18:1/22:0(2OH))	C_46_H_88_NO_12_-
883.5327	883.5342	1.65	PI(38:5)	C_47_H_80_O_13_P-
885.5502	885.5499	−0.34	PI(38:4)	C_47_H_82_O_13_P-
888.6237	888.624	0.3	SulfoHex-Cer(d18:1/24:1)	C_48_H_92_NO_11_-
890.6399	890.6397	−0.29	SulfoHex-Cer(d18:1/24:0)	C_48_H_92_NO_11_-
965.516	965.5162	0.18	PI(38:4)	C_47_H_83_O_16_P-

^a^Identifications made by mass accuracy alone

**Figure 2:**
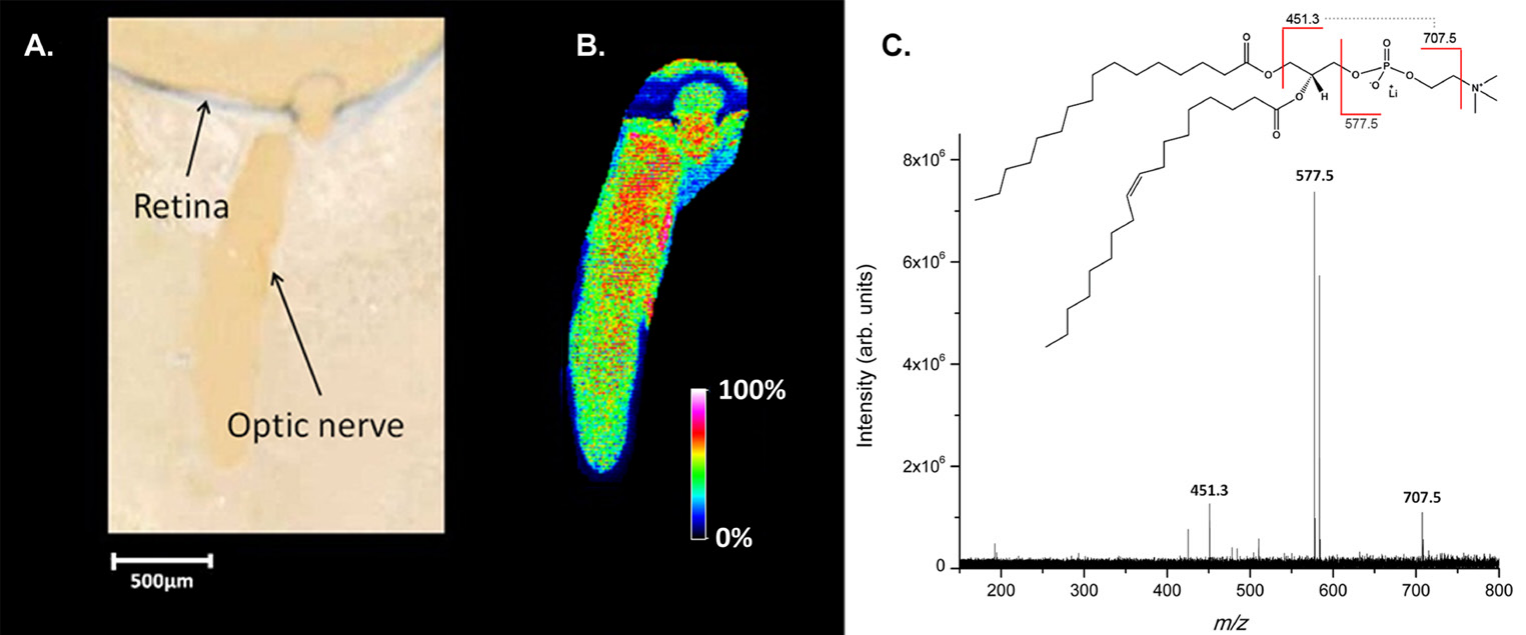
Optical image of tissue and mass spectrometry data of major lipid at m/z 760.6 from mouse optic nerve. **A**: Optical image of ocular tissue section coated with the 2,5-dihydroxybenzoic acid matrix. **B**: Matrix-assisted laser desorption ionization–imaging mass spectrometry (MALDI-IMS) image in positive ion mode at 10 µm spatial resolution of a mouse optic nerve section illustrating the distribution and intensity of a protonated phosphatidylcholine lipid at m/z 760.6. **C**: Product ion spectrum of the lithiated form of m/z 760.6 (m/z 766.6) produced from Fourier transform ion cyclotron resonance mass spectrometer analysis identified as C_42_H_82_NO_8_P (PC(16:0/18:1)).

Figure 2A displays an optical image of the region of the mouse optic nerve head and optic nerve in positive ion mode after sublimation of the DHB matrix. Figure 2B shows the distribution of a putative protonated lipid at m/z 760.6 acquired in the positive ion mode. The MALDI image indicates that this molecular species is localized in the central neuronal tissue of the optic nerve with a higher abundance toward the retina. This ion has been identified as a phosphatidylcholine (PC(16:0/18:1) using mass accuracy (0.12 ppm, Table 1) and tandem mass spectrometry as highlighted in Figure 2C. The lithiated form of this ion (m/z 766.6) was selected and accumulated using CASI allowing for high-quality SORI-CID data to be collected. The complete list of observed fragments can be found in Table 3. The observed neutral losses of trimethylamine (m/z 707.5, [M-59+Li]^+^), ethylene phosphate (m/z 583.5, [M-183+Li]^+^), and lithium ethylene phosphate (m/z 577.5, [M-183+H]^+^) are characteristic peaks associated with all glycerophosphocholines [33,34]. Additionally, for lithiated species, the fatty acid in the *sn*-1 position is known to be preferentially lost over the *sn*-2 fatty acyl chain during fragmentation [33,34]. The most abundant fragment ion associated with the loss of a fatty acyl chain was m/z 451.3 ([M-59-(16:0)+Li]+) suggesting that palmitic acid (16:0) is in the *sn*-1 position.

**Table 3:** Summary of tandem mass spectra data for selected ion at m/z 766.6^a^, PC(16:0/18:1)Li^+^.

Observed m/z	Identification	Molecular Formula	Theoretical m/z
425.266	[M-59-(18:1)+Li]+	C_21_H_39_LiO_6_P+	425.264
451.282	[M-59-(16:0)+Li]+	C_23_H_41_LiO_6_P+	451.280
478.331	[M-(18:1)+H]+	C_24_H_49_NO6P+	478.329
484.340	[M-(18:1)+Li]+	C_24_H_48_LiNO_6_P+	484.337
510.357	[M-(16:0)+Li]+	C_26_H_50_LiNO_6_P+	510.353
577.524	[M-183+H]+	C_37_H_69_O_4_+	577.534
583.532	[M-183+Li]+	C_37_H_68_LiO_4_+	583.527
707.527	[M-59+Li]+	C_39_H_73_LiO_8_P+	707.520
766.607 (isolated mass)	[M+Li]+	C_42_H_82_LiNO_8_P+	766.593

^a^ +6 Mass unit adjustment is made for the formation of a lithium adduct as a result of adding lithium chloride to the samples before identification.

**Figure 3:**
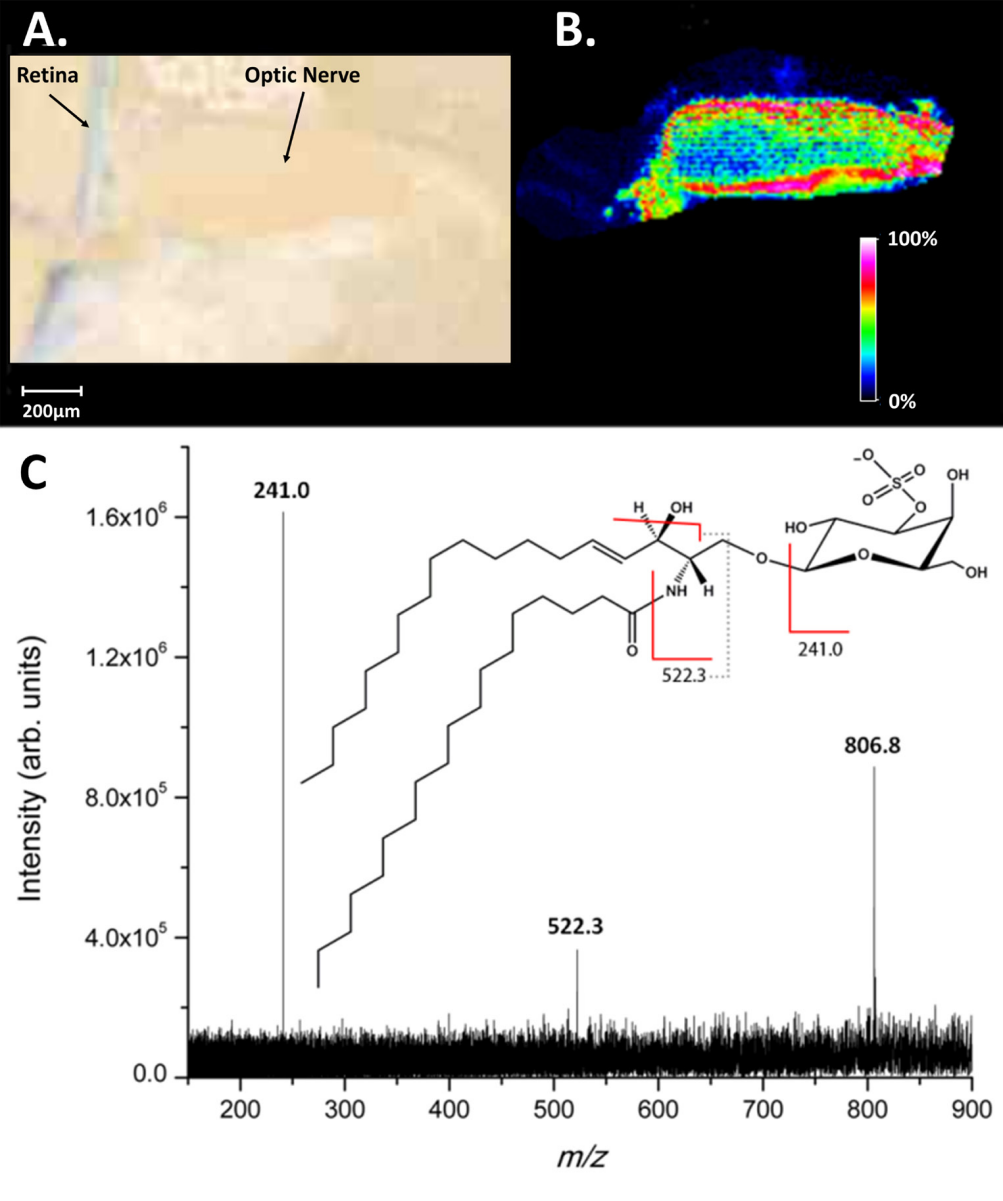
Optical image of tissue and mass spectrometry data of major lipid at m/z 806.5 from mouse optic nerve. **A**: Optical image of ocular tissue section coated with the 2,5-dihydroxybenzoic acid matrix. **B**: Matrix-assisted laser desorption ionization–imaging mass spectrometry (MALDI-IMS) image showing the distribution of the deprotonated sphingolipid species at m/z 806.5. **C**: product ion spectrum of m/z 806.5 identified as C_42_H_80_NO_11_S (Sulfatide (d18:1/18:0)).

Figure 3A shows the optical image of a mouse optic nerve section after sublimation from which data were acquired in negative ion mode. Figure 3B shows the distribution of a putative deprotonated lipid at m/z 806.5. The MALDI image shows that this molecular species is highly abundant in the meninges surrounding the nerve bundle, with lower abundance in the central neuronal tissue. Figure 3C shows the tandem mass spectrum of m/z 806.5 indicating three major fragment ions (Table 4). Based on the accurate mass measurements (0.02 ppm, Table 2) and fragmentation patterns, the deprotonated species was identified as sulfatide(d18:1/18:0). The most abundant fragment ion (m/z 241.0) is related to the 3-sulfogalactosyl moiety, and the minor fragment (m/z 522.3) results from the loss of the fatty acyl chain as a ketene followed by loss of water. Both are common fragmentation pathways of sulfatides [35]. Sulfatides account for around 4%–7% of central nervous system myelin by weight and have been shown to be involved in cell signaling pathways and inhibiting axon regeneration [36].

**Table 4:** Summary of tandem mass spectra data for selected ion at m/z 806.5, sulfatide (d18:1/18:0).

Observed m/z	Identification	Molecular Formula	Theoretical m/z
240.997	[(3′-sulfo)Galβ-H]-	C_6_H_9_O_8_S-	241.002
522.271	[M-(18:0)-H]-	C_24_H_44_NO_9_S-	522.274
806.554	[M-H]-	C_42_H_80_NO_11_S-	806.546

**Figure 4:**
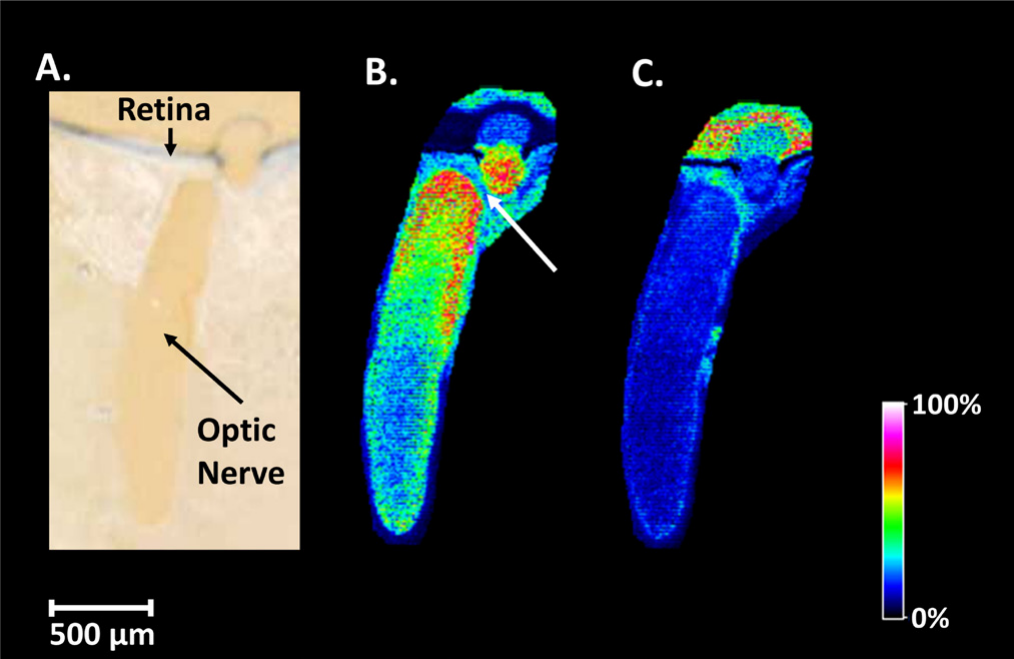
Optical and mass spectrometry images of mouse optic nerve tissue. **A**: Optical image of mouse ocular tissue section coated with the 2,5-dihydroxybenzoic acid matrix. **B**: Matrix-assisted laser desorption ionization–imaging mass spectrometry (MALDI-IMS) image in positive ion mode at 10 µm spatial resolution of a mouse optic nerve illustrating the distribution and intensity of a protonated phosphatidylcholine species C_44_H_86_NO_8_P (PC(18:1/18:0)) at m/z 788.6. **C**: MALDI-IMS image illustrating the distribution and intensity of a protonated phosphatidylcholine species C_40_H_80_NO_8_P (PC (16:0:16:0)) at m/z 734.6.

Figure 4A shows an optical image of the region of the mouse optic nerve head and optic nerve imaged after the DHB matrix was applied via sublimation. MALDI imaging data were acquired in positive ion mode, and Figure 4B shows the distribution of the protonated phosphatidylcholine species at m/z 788.6 identified as PC (18:0/18:1; Table 5). The MALDI image shows that this species is localized in the optic nerve head and at the anterior of the optic nerve within the central neuronal tissue. The region of low intensity between the optic nerve head and the optic nerve fiber is an artifact of sectioning, i.e., the nerve fiber was not perfectly aligned within the ice-embedded tissue. The slight kink in the nerve fiber that naturally precedes insertion into the retina resulted in no nerve tissue being present in the region indicated by the white arrow. Figure 4C shows the distribution of the protonated phosphatidylcholine species (PC(16:0/16:0) Table 6) at m/z 734.6; the MALDI image shows that this species is located in the optic nerve head.

**Table 5:** Summary of tandem mass spectral data for selected ion at m/z 794.6^a^, PC(18:0/18:1)Li^+^.

Observed m/z	Identification^a^	Molecular Formula	Theoretical m/z
451.283	[M-59-(18:0)+H]+	C_23_H_41_LiO_6_P+	451.280
506.364	[M-(18:1)+H]+	C_26_H_53_NO_6_P+	506.361
510.355	[M-(18:0)+Li]+	C_26_H_50_LiNO_6_P+	510.353
605.555	[M-183+H]+	C_39_H_73_O_4_+	605.550
611.564	[M-183+Li]+	C_39_H_72_LiO_4_+	611.559
735.560	[M-59+Li]+	C_41_H_77_LiO_8_P+	735.551
794.638	[M+Li]+	C_44_H_86_LiNO_8_P+	794.625

^a^ +6 Mass unit adjustment is made for the formation of a lithium adduct as a result of adding lithium chloride to the samples before identification.

**Table 6:** Summary of tandem mass spectral data for selected ion at m/z 740.6^a^, PC(16:0/16:0)Li^+^.

Observed m/z	Identification^a^	Molecular Formula	Theoretical m/z
425.266	[M-59-(16:0)+Li]+	C_21_H_39_LiO_6_P+	425.264
551.507	[M-183+H]+	C_35_H_67_O_4_+	551.503
557.518	[M-183+Li]+	C_35_H_66_LiO_4_+	557.512
740.59	[M+Li]+	C_40_HLiNO_8_P+	740.578

^a^ +6 Mass unit adjustment is made for the formation of a lithium adduct as a result of adding lithium chloride to the samples before identification.

**Figure 5:**
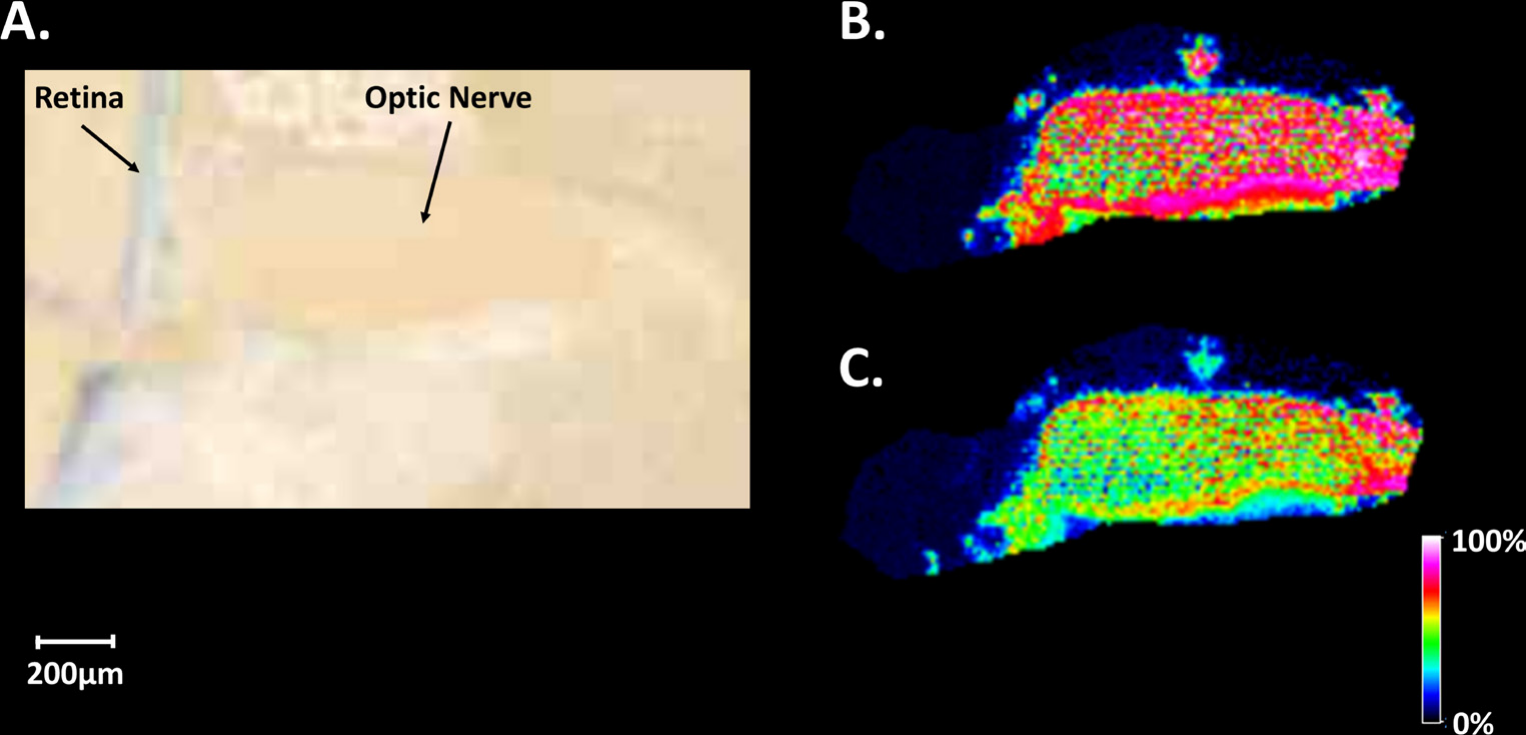
Optical and mass spectrometry images of mouse optic nerve tissue. **A**: Optical image of mouse ocular tissue section coated with the 2,5-dihydroxybenzoic acid matrix. **B**: Matrix-assisted laser desorption ionization–imaging mass spectrometry (MALDI-IMS) image in negative ion mode at 10 µm spatial resolution of a mouse optic nerve illustrating the distribution and intensity of a deprotonated sphingolipid species C_48_H_92_NO_11_S sulfatide (d18:1:24:0)) at m/z 890.6. **C**: MALDI-IMS image illustrating the distribution and intensity of a deprotonated sphingolipid species C_46_H_88_NO_12_S (Sulfatide(d18:0/22:0(2OH)) at m/z 878.6.

Figure 5A shows an optical image of the region of the mouse optic nerve head and optic nerve imaged after the matrix was applied via sublimation. MALDI imaging data were acquired in negative ion mode, and Figure 5B shows the distribution of a deprotonated sphingolipid species at m/z 890.6 with the molecular formula C_48_H_92_NO_11_S, sulfatide (d18:1/24:0; Table 7). This sulfatide has a high abundance throughout the majority of the neuronal tissue, with the highest abundance in the meninges surrounding the optic nerve. Figure 5C shows the distribution of another deprotonated sphingolipid species at m/z 878.6 with the molecular formula C_46_H_88_NO_12_S, sulfatide (d18:0/22:0(2OH); Table 8). The MALDI image shows that this species is present with lower abundance than sulfatide (d18:1/24:0) and is predominantly observed in the meninges surrounding the nerve fiber away from the retina and optic nerve head.

**Table 7:** Summary of tandem mass spectral data for selected ion at m/z 890.7, Sulfatide(d18:1/24:0).

Observed m/z	Identification^a^	Molecular Formula	Theoretical m/z
240.997	[(3′-sulfo)Galβ-(H2O)-H]-	C_6_H_9_O_8_S-	241.002
522.279	[M-(24:0)-H]-	C_24_H_44_NO_9_S-	522.274
890.649	[M-H]-	C48H92NO11S-	890.640

**Table 8:** Summary of tandem mass spectral data for selected ion at m/z 878.6, Sulfatide(d18:0/22:0(2OH)).

Observed m/z	Identification^a^	Molecular Formula	Theoretical m/z
240.997	[(3′-sulfo)Galβ-(H2O)-H]-	C_6_H_9_O_8_S-	241.002
259.007	[(3′-sulfo)Galβ-H]-	C_6_H_11_O_9_S-	259.013
507.266	[M-(22:0(OH))-(OH)-(NH3)-H]-	C_24_H_43_O_9_S-	507.263
522.277	[M-(22:0(OH))-(OH)-H]-	C_24_H_44_NO_9_S-	522.274
540.289	[M-(22:0(OH))-H]-	C_24_H_46_NO_10_S-	540.285
568.284	[M-(C21H42O)-H]-	C_25_H_46_NO_11_S-	568.28
878.618	[M-H]-	C_46_H_88_NO_12_S-	878.603

**Figure 6:**
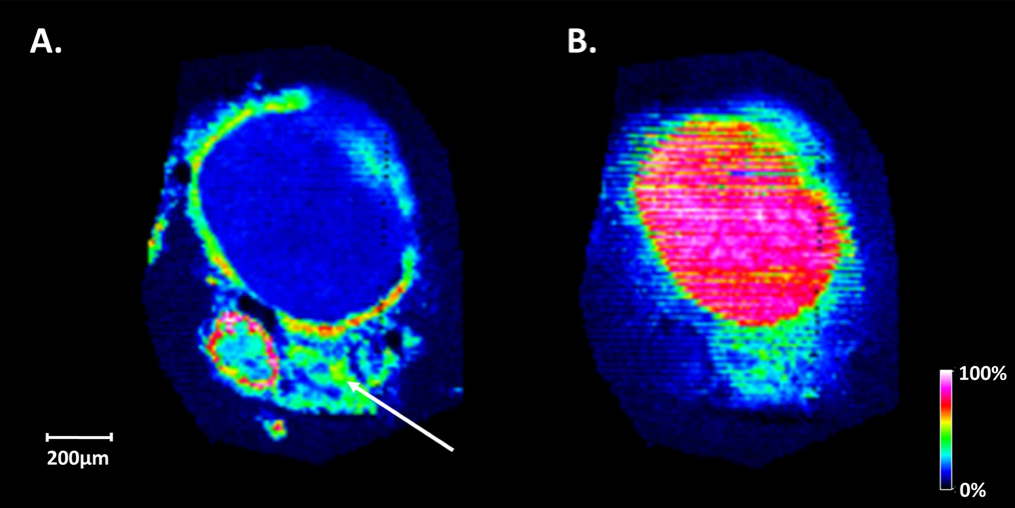
Mass spectrometry images of rat optic nerve tissue. **A**: Matrix-assisted laser desorption ionization–imaging mass spectrometry (MALDI-IMS) image showing the distribution of PC(18:0/20:4) at m/z 810.6 located in the meninges, connective tissue, and the wall of a blood vessel running parallel to the optic nerve fiber. **B**: MALDI-IMS image of PC(18:0/18:1) at m/z 788.6 located in the optic nerve.

**Figure 7:**
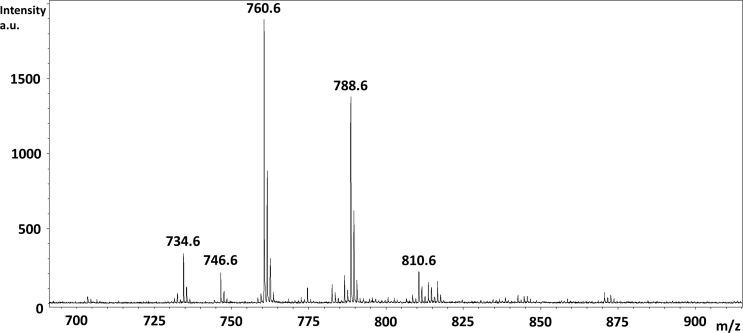
Matrix-assisted laser desorption ionization mass spectrum from rat optic nerve tissue. Positive ion mode matrix-assisted laser desorption ionization–time-of-flight (MALDI-TOF) mass spectrum from rat optic nerve tissue showing numerous peaks in the m/z 700–875 region.

To image coronal sections, a slightly larger rat optic nerve was examined. Figure 6A shows the distribution of a protonated lipid at m/z 810.6 (PC(18:0/20:4; Table 9) in a cross section from a rat optic nerve fiber with high abundance present in the optic nerve sheath, in the wall of a vein alongside the optic nerve fiber and a lower abundance present in the meninges surrounding the optic nerve and the connective tissue between the two (indicated by the arrow). Figure 6B shows the distribution of a protonated lipid at m/z 788.6 (PC(18:0/18:1; Table 10) in the central region of the optic nerve with high abundance throughout the tissue. Figure 7 shows a positive ion MALDI mass spectrum from this tissue.

**Table 9:** Summary of tandem mass spectral data for selected ion at m/z 816.6^a^, PC(18:0/20:4)Li^+^.

Observed m/z	Identification^a^	Molecular Formula	Theoretical m/z
532.338	[M-(18:0)+Li]+	C_21_H_39_LiO_6_P+	532.337
633.546	[M-183+Li]+	C_41_H_70_LiO_4_+	633.543
757.539	[M-59+Li]+	C_43_H_75_LiO_8_+	757.535
816.612	[M+Li]+	C_44_H_86_LiNO_8_P+	816.609

^a^ +6 Mass unit adjustment is made for the formation of a lithium adduct as a result of adding lithium chloride to the samples before identification.

**Table 10:** Summary of tandem mass spectral data for selected ion at m/z 794.636^a^, PC(18:0/18:1)Li^+^.

Observed m/z	Identification^a^	Molecular Formula	Theoretical m/z
451.282	[M-59-(18:0)+H]+	C_23_H_41_LiO_6_P+	451.28
506.361	[M-(18:1)+H]+	C_26_H_53_NO_6_P+	506.361
510.354	[M-(18:0)+Li]+	C_26_H_50_LiNO_6_P+	510.353
605.554	[M-183+H]+	C_39_H_73_O_4_+	605.55
611.563	[M-183+Li]+	C_39_H_72_LiO_4_+	611.559
735.559	[M-59+Li]+	C_41_H_77_LiO_8_P+	735.551
794.636	[M+Li]+	C_44_H_86_LiNO_8_P+	794.625

^a^ +6 Mass unit adjustment is made for the formation of a lithium adduct as a result of adding lithium chloride to the samples before identification.

## Discussion

Using high spatial resolution (10×10 μm) MALDI imaging mass spectrometry, we demonstrated localized and identified lipid species and their relative abundances within small structures of mouse and rat optic nerve tissue of 200–600 μm diameter. The sample preparation method described allowed images to be acquired at 10 μm spatial resolution. Images acquired in positive ion mode provided the distribution of several phosphatidylcholine species and one sphingomyelin associated with the internal axon fibers and surrounding meninges of the optic nerve (Table 1 and Appendix 1). The data observed in negative ion mode indicated several sulfatides along with other lipid species including phosphoinositols and a phosphoinositol monophosphate (Table 2 and Appendix 2) with the highest abundance within the meninges surrounding the nerve, the internal axon fibers, and the optic nerve head region. Murphy et al. [37] demonstrated that these lipid species are expected when using DHB as a matrix. An alternative matrix such as 1,5-diaminonaphthalene has been shown to provide excellent sensitivity at high spatial resolutions and more variety toward lipid species [25] that could be amenable to this tissue type and disease model tissue. As optic nerve tissue is regarded as an extension of the white matter of the brain, these lipid species have been previously described in several MALDI-IMS, time-of-flight secondary ion mass spectrometry (TOF-SIMS), and electrospray ionization mass spectrometry (ESI-MS) publications from spinal cord tissue and rodent brain [36,38-40]. The data presented demonstrate the potential use of MALDI-IMS to compare lipid composition, abundance, and localization in optic nerve tissue in various disease models.

Although not much is known about the functionality of lipids in the central nervous system, the localization of protein and lipid changes within the optic nerve and retina in models of glaucoma is relevant to pathology. Degeneration of retinal ganglion cell bodies and their axons is not uniform, but rather occurs in a sectorial pattern from one retinotopic region to the next. Wedge- or fan-shaped regions of degeneration have been observed in the DBA/2J mouse model of glaucoma, as well as in different acute models [9,41-44]. These sectorial losses in the retina also correspond to regional loss of retinal ganglion cell axons within the optic nerve [8,45] and to focal deficits in axonal transport within the superior colliculus [7,46]. Interestingly, the sectorial pattern of retinal ganglion cell degeneration has also been observed in human glaucomatous retinas [47].

The method of sublimation to apply the matrix provides a small crystal in a dense lattice. This property combined with removing the biologic salts from the tissue sections with the wash step, provided sufficient sensitivity allowing for a small (<10 µm) laser spot to be used. This method allows for reliable imaging analysis and identification of endogenous lipids within small tissue structures. Selective ion accumulation in the hexapole region of the FT-ICR using the CASI method before fragmentation made lipid identification easier as the signal intensity for the fragments were significantly improved. In conclusion, the MALDI-IMS technology shows great promise for generating spatial and molecular information in diseases affecting optic nerve structure and function, leading to information that can be used to understand molecular mechanisms of disease and to develop new therapeutic strategies.

## Appendix 1.

Matrix-assisted laser desorption ionization–imaging mass spectrometry images of selected lipids observed from mouse optic nerve tissue in positive ion mode. Lipid assignments were made based on mass accuracy alone. To access the data, click or select the words “Appendix 1.”

## Appendix 2.

Matrix-assisted laser desorption ionization–imaging mass spectrometry images of selected lipids from mouse optic nerve tissue observed in negative ion mode. Lipid assignments were based on mass accuracy alone. To access the data, click or select the words “Appendix 2.”
